# The Association of the Vanin-1 N131S Variant with Blood Pressure Is Mediated by Endoplasmic Reticulum-Associated Degradation and Loss of Function

**DOI:** 10.1371/journal.pgen.1004641

**Published:** 2014-09-18

**Authors:** Ya-Juan Wang, Bamidele O. Tayo, Anupam Bandyopadhyay, Heming Wang, Tao Feng, Nora Franceschini, Hua Tang, Jianmin Gao, Yun Ju Sung, the COGENT BP consortium, Robert C. Elston, Scott M. Williams, Richard S. Cooper, Ting-Wei Mu, Xiaofeng Zhu

**Affiliations:** 1 Department of Epidemiology and Biostatistics, School of Medicine, Case Western Reserve University, Cleveland, Ohio, United States of America; 2 Center for Proteomics and Bioinformatics, School of Medicine, Case Western Reserve University, Cleveland, Ohio, United States of America; 3 Department of Public Health Sciences, Loyola University Chicago, Stritch School of Medicine, Maywood, Illinois, United States of America; 4 Department of Chemistry, Boston College, Chestnut Hill, Massachusetts, United States of America; 5 Department of Epidemiology, University of North Carolina at Chapel Hill, Chapel Hill, North Carolina, United States of America; 6 Department of Genetics, Stanford University School of Medicine, Stanford, California, United States of America; 7 Division of Biostatistics, Washington University School of Medicine, St Louis, Missouri, United States of America; 8 Department of Genetics, Geisel School of Medicine, Dartmouth College, Hanover, New Hampshire, United States of America; 9 Department of Physiology and Biophysics, School of Medicine, Case Western Reserve University, Cleveland, Ohio, United States of America; The University of North Carolina at Chapel Hill, United States of America

## Abstract

High blood pressure (BP) is the most common cardiovascular risk factor worldwide and a major contributor to heart disease and stroke. We previously discovered a BP-associated missense SNP (single nucleotide polymorphism)–rs2272996–in the gene encoding vanin-1, a glycosylphosphatidylinositol (GPI)-anchored membrane pantetheinase. In the present study, we first replicated the association of rs2272996 and BP traits with a total sample size of nearly 30,000 individuals from the Continental Origins and Genetic Epidemiology Network (COGENT) of African Americans (P = 0.01). This association was further validated using patient plasma samples; we observed that the N131S mutation is associated with significantly lower plasma vanin-1 protein levels. We observed that the N131S vanin-1 is subjected to rapid endoplasmic reticulum-associated degradation (ERAD) as the underlying mechanism for its reduction. Using HEK293 cells stably expressing vanin-1 variants, we showed that N131S vanin-1 was degraded significantly faster than wild type (WT) vanin-1. Consequently, there were only minimal quantities of variant vanin-1 present on the plasma membrane and greatly reduced pantetheinase activity. Application of MG-132, a proteasome inhibitor, resulted in accumulation of ubiquitinated variant protein. A further experiment demonstrated that atenolol and diltiazem, two current drugs for treating hypertension, reduce the vanin-1 protein level. Our study provides strong biological evidence for the association of the identified SNP with BP and suggests that vanin-1 misfolding and degradation are the underlying molecular mechanism.

## Author Summary

Hypertension (HTN) or high blood pressure (BP) is common worldwide and a major risk factor for cardiovascular disease and all-cause mortality. Identification of genetic variants of consequence for HTN serves as the molecular basis for its treatment. Using admixture mapping analysis of the Family Blood Pressure Program data, we recently identified that the *VNN1* gene (encoding the protein vanin-1), in particular SNP rs2272996 (N131S), was associated with BP in both African Americans and Mexican Americans. Vanin-1 was reported to act as an oxidative stress sensor using its pantetheinase enzyme activity. Because a linkage between oxidative stress and HTN has been hypothesized for many years, vanin-1's pantetheinase activity offers a physiologic rationale for BP regulation. Here, we first replicated the association of rs2272996 with BP in the Continental Origins and Genetic Epidemiology Network (COGENT), which included nearly 30,000 African Americans. We further demonstrated that the N131S mutation in vanin-1 leads to its rapid degradation in cells, resulting in loss of function on the plasma membrane. The loss of function of vanin-1 is associated with reduced BP. Therefore, our results indicate that vanin-1 is a new candidate to be manipulated to ameliorate HTN.

## Introduction

Hypertension (HTN) or high blood pressure (BP) is common in populations worldwide and a major risk factor for cardiovascular disease (CVD) and all-cause mortality [1]. Although it is observed across ethnically diverse populations, the prevalence of HTN in the US varies from 27% in persons of European ancestry to 40% among those of African ancestry [2]. BP is a moderately heritable trait and affected by the combined effects of genetic and environmental factors, with heritable factors cumulatively accounting for 30–55% of the variance [3]. After age 20, African Americans have higher BP than other US race/ethnicities [4]–[6] and the progression from pre-HTN to HTN occurs one year eariler on average [7]. Increased rates of HTN among African Americans are the main factor contributing to their greater risk of CVD and end-stage renal disease compared to US whites [8], [9]. Given the widespread occurrence of this condition, and our as yet limited ability to reduce the disease burden, identifying the genetic variants of BP phenotypes could elucidate the underlying biology of high BP and reduce the CVD prevalence.

Identification of genetic variants of consequence for HTN remains a significant challenge, owing in large part to the complex and polygenic nature of the disorder and the imprecision with which the phenotype is measured [10]. Using admixture mapping analysis of data from the Family Blood Pressure Program, we recently identified a genomic region on chromosome 6 harboring HTN-associated variants [11]. The same region on chromosome 6 was replicated in an admixture mapping analysis based on the African Americans enrolled in the Dallas Heart Study [12]. By further genotyping the functional variants in the region of interest on chromosome 6, the *VNN1* gene, in particular SNP rs2272996 (N131S) was found to account for the association with BP in both African Americans and Mexican Americans, but this association was not observed in European Americans [12]. Fava et al. [13] recently argued that rs2294757 (T26I), rather than N131S, was a more likely functional variant accounting for the effect on BP because it is located in a splicing regulation site in *VNN1*, but these investigators only found a weak association between T26I and both DBP and HTN in one of the two studies that they carried out. The results of this study are consistent with the lack of evidence for association observed in European Americans in the Dallas Heart Study [12].

*VNN1* encodes the protein vanin-1, a glycosylphosphatidylinositol (GPI)-anchored membrane protein [14], [15]. Vanin-1 is widely expressed in a variety of tissues, with higher expression in liver, kidney and blood [16]. Vanin-1 is a pantetheinase, a member of the biotinidase branch of the nitrilase superfamily [17]. Vanin-1 hydrolyzes pantetheine to pantothenic acid (vitamin B5) and cysteamine, a potent regulator of oxidative stress. In vanin-1 null mice free cysteamine is undetectable, indicating vanin-1's indispensable role in generating cysteamine under physiological conditions [18]. Therefore, vanin-1 plays an essential role in regulating oxidative stress via cysteamine generation. A linkage between oxidative stress and HTN has been hypothesized for many years [19]–[21]. Furthermore, vanin-1 was reported to be involved in cardiovascular diseases [22], [23]. Overexpression of vanin-1 was associated with progression to chronic pediatric immune thrombocytopenia (ITP) [24], and was shown to lead to hyperglycemia [25]. Vanin-1^−/−^ mice showed protective effects against a variety of phenotypes, such as oxidative stress [26], intestinal inflammation [27], and colon cancer [28], mostly due to higher glutathione storage to maintain a more reducing environment. As a consequence, vanin-1's pantetheinase activity may offer a physiologic rationale for BP regulation with loss of vanin-1 function.

In this study, we first investigated the association evidence of the missense variant rs2272996 (N131S) in *VNN1* and BP phenotypes by performing a meta-analysis of nearly 30,000 African ancestry subjects from 19 independent cohorts from the Continental Origins and Genetic Epidemiology Network (COGENT). We next examined whether there were other variants in *VNN1* associated with BP traits. Lastly, we conducted molecular experiments to establish a functional connection between N131S vanin-1 and HTN.

## Results

### Meta-analysis of *VNN1* with BP

**Figure 1:**
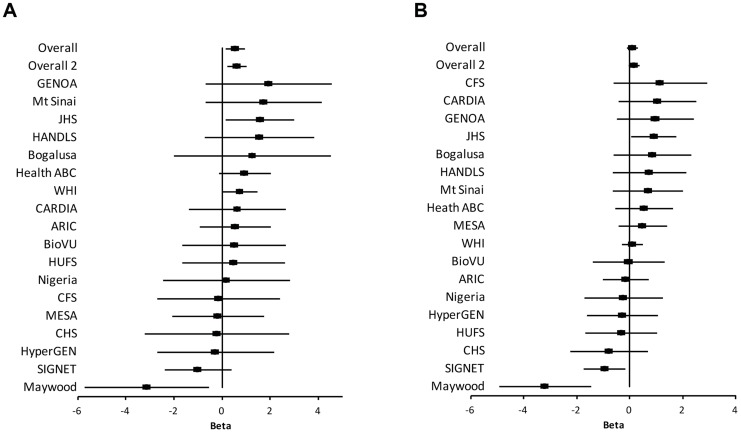
Association of SNP rs2272996 with SBP (A) and DBP (B) for each cohort. The effect size (Beta) and 95% confidence interval are presented in terms of the reference allele T. Overall: all COGENT cohorts are included in the meta-analysis. Overall 2: Maywood cohort is excluded from the meta-analysis. Biological Bank of Vanderbilt University (BioVU); Atherosclerosis Risk In Communities (ARIC); Coronary Artery Risk Development in Young Adults (CARDIA); Cleveland Family Study (CFS); Jackson Heart Study (JHS); Multi-Ethnic Study of Atherosclerosis (MESA); Cardiovascular Health Study (CHS); Genetic Study of Atherosclerosis Risk (GeneSTAR); Genetic Epidemiology Network of Arteriopathy (GENOA); The Healthy Aging in Neighborhoods of Diversity Across the Life Span Study (HANDLS); Health, Aging, and Body Composition (Health ABC) Study; The Hypertension Genetic Epidemiology Network (HyperGEN); Mount Sinai, New York City, USA Study (Mt Sinai Study); Women's Health Initiative SNP Health Association Resource (WHI); Howard University Family Study (HUFS); Bogalusa Heart Study (Bogalusa); Sea Islands Genetic Network (SIGNET); Loyola Maywood Study (Maywood); and Loyola Nigeria Study (Nigeria).

The study samples were the African-ancestry subjects from the COGENT, which includes 19 discovery cohorts. The details are described elsewhere by Franceschini et al [29]. Briefly, the phenotype-genotype association analysis was performed in each cohort separately. Systolic BP (SBP) and diastolic BP (DBP) were treated as continuous variables. For individuals reporting the use of antihypertensive medications, BP was adjusted by adding 10 and 5 mmHg to SBP and DBP respectively [30]. SBP and DBP were adjusted for age, age^2^, body mass index (BMI) and gender in linear regression models. The results of association between SNP rs2272996 and SBP or DBP for the 18 cohorts are presented in **Figure 1**. This SNP was not available in the GeneSTAR cohort. The corresponding allele frequencies in the different studies are listed in Supplementary **Table S1**. Among the 18 cohorts, 12 and 10 have positive effect sizes for SBP (P = 0.048) and DBP (P = 0.24), respectively, comparing to 9 expected under null hypothesis of no association between this SNP and BP. We next performed meta-analysis by applying both fixed-effect [31], [32] and random-effect [33] models to estimate the overall effect. SNP rs2272996 was significantly associated with SBP in both fixed-effect (P = 0.01) and random-effect (P = 0.04) models (**Table 1**). However, we did not observe evidence of genotype-phenotype association for DBP.

**Table 1:** Meta-analysis results of the COGENT cohorts data for SNP rs2272996 (N131S).

						Fixed-effects Model			Random-effects Model		
Cohorts	Phenotype	A1	Frequency	Direction in each cohort	Heterogeneity *p* value	Beta (95% CI)	SE	*p* value	Beta (95% CI)	SE	*p* value
Overall^a^	SBP	T	0.81	++++−−?++++−+−−+−++	0.28	0.52 (0.12, 0.91)	0.20	**0.010**	0.48 (0.02, 0.93)	0.23	**0.040**
Excluding Maywood^b^	SBP	T	0.81	++++−−?++++−+−+−++	0.75	0.60 (0.21, 1.0)	0.20	**0.003**	0.60 (0.21, 1.0)	0.20	**0.003**
Overall^a^	DBP	T	0.81	−−+++−?+++−−+−+−−++	0.01	0.09 (−0.14, 0.32)	0.1	0.45	0.10 (−0.28,0.48)	0.19	0.60
Excluding Maywood^b^	DBP	T	0.81	−−+++−?+++−−++−−++	0.19	0.15 (−0.08,0.38)	0.1	0.21	0.18 (−0.11,0.48)	0.15	0.22

The effect size (beta) is presented in terms of the reference allele A1 for the SNP of interest. SE: standard error. CI: confidence interval.

a All COGENT cohorts were included in the meta-analysis. This SNP (rs2272996) was not available in GeneSTAR.

b Maywood cohort was excluded from the meta-analysis.

Among the individual cohort analyses, the Maywood cohort had a sample size of 743 and was the only cohort that showed significant association with rs2272996 for both SBP (P = 0.016) and DBP (P = 0.0003), however the direction of the association was opposite to what was found in the test for overall effects (**Figure 1**). The distributions of SBP, DBP, age and BMI did not suggest that the Maywood was an outlier in epidemiologic characteristics (Supplementary **Figure S1**), with the exception that the sampling strategy for this cohort was based on exclusion of persons on antihypertensive medications (antihypertensive medication rate was 0.7%). The Nigeria cohort also included a low antihypertensive medication rate but this was a result of inaccessibility to medications (Supplementary **Figure S2**). When analyses were repeated after exclusion of the Maywood cohort, the association of BP and rs2272996 was substantially improved (P = 0.003 for SBP, **Table 1**). The different association evidence between Maywood and other cohorts may suggest genetic heterogeneity or possible interaction between gene and environment factors, although further studies are needed to address this possibility.

### Searching for additional BP variants in *VNN1*

Since additional genetic variants in *VNN1* might be associated with BP, we examined available known variants in *VNN1* including 10 kb up- and down-stream of the gene. A total of 105 other SNPs were available in the 19 cohorts. SNP rs7739368 had the smallest *p* values for association with SBP using either fixed-effect model (P = 0.004) or random-effect model (P = 0.004, Supplementary **Table S2**), but this was not significant after correcting for multiple comparisons. This SNP is ∼7 k bp's upstream of *VNN1* adjacent to the PU.1 transcription factor binding region (306 bp's upstream).

### The vanin-1 expression in human plasma samples is closely linked with both genotypic N131S mutation and phenotypic HTN

**Figure 2:**
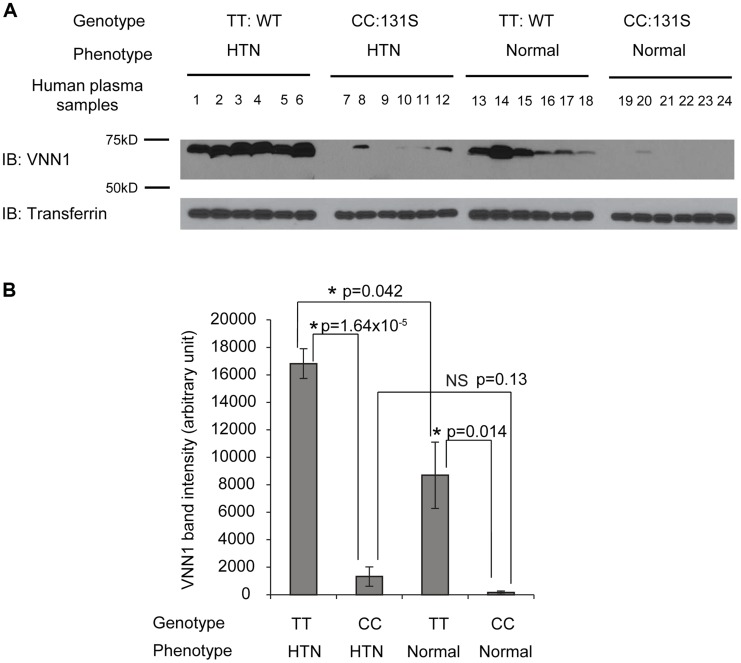
The vanin-1 expression in human plasma samples is closely linked with both genotypic N131S mutation and phenotypic HTN. Human plasma samples were collected from 6 HTN patients with WT *VNN1* (samples 1 to 6), 6 HTN patients with homozygous N131S *VNN1* (samples 7 to 12), 6 healthy controls with WT *VNN1* (samples 13 to 18), and 6 healthy controls with homozygous N131S *VNN1* (samples 19 to 24). Same amount of total plasma proteins were subjected to 8% SDS-PAGE and Western blot analysis; transferrin serves as loading control. (**A**). IB: immunoblotting. WT: wild type. The vanin-1 protein band intensity was quantified using ImageJ software from the NIH, shown in (**B**). NS: not significant. Data are reported as mean ± SEM.

To understand the function of N131S vanin-1 in relation to HTN, plasma samples from Nigeria HTN patients and normotensives with WT (TT) or homozygous N131S (CC) vanin-1 were collected (6 samples per group, 4 groups). The same amount of total plasma protein from each sample was subjected to Western blot analysis: a clean vanin-1 protein band appeared at 70 kD (**Figure 2A**), consistent with previous reports [14], [34]. The plasma vanin-1 protein in homozygous N131S vanin-1 was significantly lower than that in WT vanin-1 in both hypertensive and normotensive groups (P = 1.64×10^−5^ and 0.014, **Figure 2A**, see **Figure 2B** for quantification), indicating that the N131S mutation is a functional variant that is associated with substantially less steady-state vanin-1 protein. Furthermore, the plasma vanin-1 protein in normotensive groups with WT vanin-1 (samples 13–18) was significantly lower than that in HTN patients with WT vanin-1 (samples 1–6) (P = 0.042) (**Figure 2A**, see **Figure 2B** for quantification). These results demonstrated that vanin-1 expression is associated with both the genotypic N131S mutation and phenotypic HTN, with the former exerting stronger effect. Lastly, the plasma vanin-1 protein in normotensive groups with homozygous N131S vanin-1 (samples 19–24) is also lower than that in HTN patients with homozygous N131S vanin-1 (samples 7–12) although it was not statistically significant (P = 0.13), probably due to the already exceedingly low vanin-1 quantity. These results suggest that the WT vanin-1 is associated with increased plasma vanin-1 protein expression, and increased HTN risk.

### N131S mutation leads to significantly lower total and surface vainin-1 protein level and fractional pantetheinase activity compared to WT and T26I vanin-1 variants

**Figure 3:**
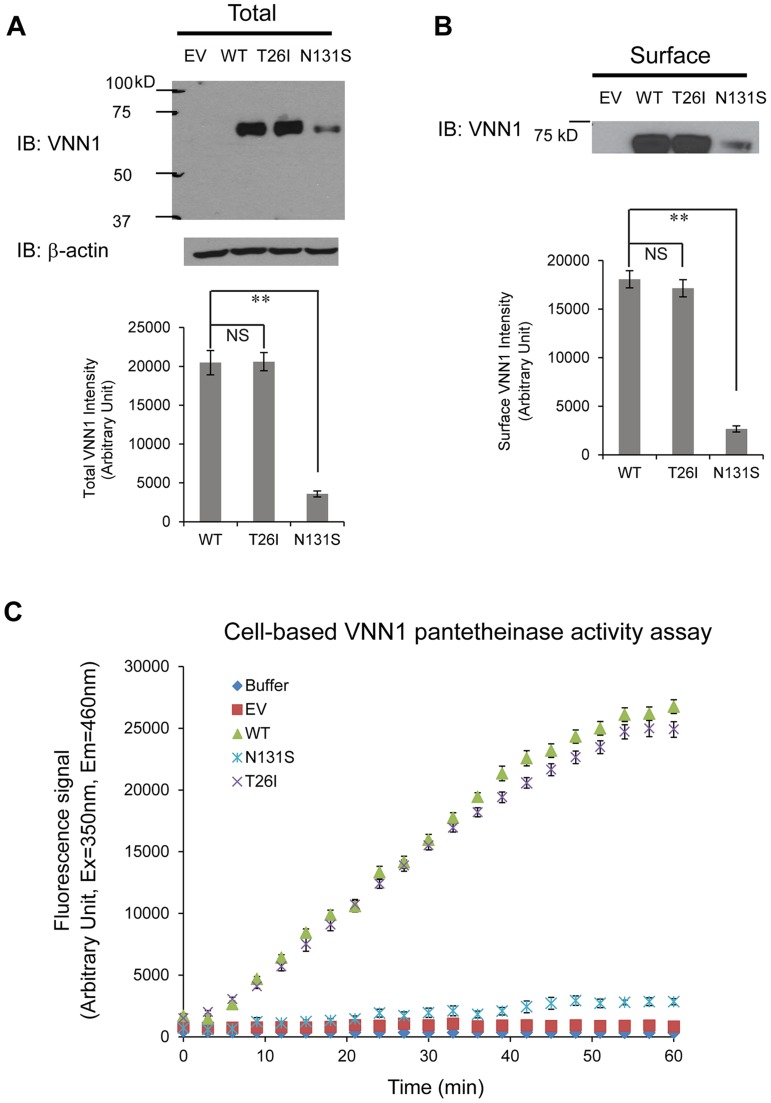
N131S mutation leads to significantly lower total and surface vainin-1 protein and fractional pantetheinase activity compared to WT, whereas T26I does not. HEK293 cells stably expressing WT, T26I or N131S vanin-1 were generated using the G418 selection method. HEK293 cells transfected with empty vector (EV) were used as a negative control. (**A**) Cells were lysed and 30 µg of total protein were subjected to 8% SDS-PAGE and Western blot analysis using a rabbit polyclonal anti-vanin-1 antibody (Pierce antibodies) (*n* = 3). β-actin serves as a loading control. (**B**) Cells were incubated with the membrane-impermeable biotinylation reagent Sulfo-NHS SS-Biotin (Pierce) and biotinylated surface proteins were affinity-purified using immobilized neutravidin-conjugated agarose beads (Pierce). Surface proteins were subjected to 8% SDS-PAGE and Western blot analysis using a rabbit polyclonal anti-vanin-1 antibody (Pierce antibodies) (*n* = 3). The protein band intensity in (**A**) and (**B**) was quantified using ImageJ software from the NIH. Data is reported as mean ± SEM. ** *p*<0.01. NS: non-significant. (**C**) A cell-based fluorescence assay was used to evaluate vanin-1 variants' pantetheinase activity according to published procedure with modifications [36]. The substrate, pantothenate-7-amino-4-methylcoumarin (Pantothenate-AMC) was chemically synthesized. Cells were lysed and 10 µg of total proteins were added to the substrate Pantothenate-AMC (5 µM) in a 100 µL final volume. Fluorescence signals at excitation 350 nm and emission 460 nm measuring the released AMC were recorded every 3 min using a fluorescence plate reader. A 60-min kinetics assay in four replicates and three biological replicates was carried out. Buffer only and HEK293 cells transfected with empty vector (EV) were used as negative controls for non-specific pantetheinase activity.

We tested two variants, N131S and T26I, as regards how they influence the total vanin-1 protein levels because other investigators have suggested that T26I may be a candidate variant for BP variation as well [13]. We utilized the human embryonic kidney 293 (HEK293) cells stably expressing these vanin-1 variants because HEK293 cells have high transfection efficiency and physiologically-relevant cell environment for vanin-1 protein expression [23]. Significantly lower total vanin-1 proteins were detected in the cells expressing N131S vanin-1, whereas similar vanin-1 protein levels were detected in cells expressing T26I vanin-1 compared to cells expressing WT vanin-1 (**Figure 3A**, quantification shown below).

Because vanin-1 is a GPI-anchored membrane protein, it needs to traffic efficiently to the plasma membrane for its pantetheinase activity. We hypothesized that N131S substantially reduces the trafficking of vanin-1 protein to the plasma membrane, whereas T26I does not. Using a surface biotinylation assay [35] , we observed that the N131S mutation led to significantly lower plasma membrane expression, whereas in cells expressing the T26I mutation, vanin-1 surface expression was similar to that observed in WT cells (**Figure 3B**, quantification shown below).

We further confirmed that the variation in vanin-1 protein expression resulted in corresponding functional consequences for N131S and T26I mutations. A cell-based fluorescence assay was carried out to record the kinetics of pantetheinase activity by vanin-1 variants [36]. The cells expressing T26I vanin-1 had similar pantetheinase activity compared to cells expressing WT vanin-1; however, cells expressing N131S vanin-1 retained approximately 9% of the pantetheinase activity, by quantifying the fluorescence signals at the kinetic steady state at 57 minutes (**Figure 3C**). These data taken together provide evidence of less protein, less membrane trafficking, and lower enzymatic activity of the N131S protein as compared to both the wild type and the T26I variant.

### N131S vanin-1 is subjected to rapid endoplasmic reticulum-associated degradation (ERAD)

**Figure 4:**
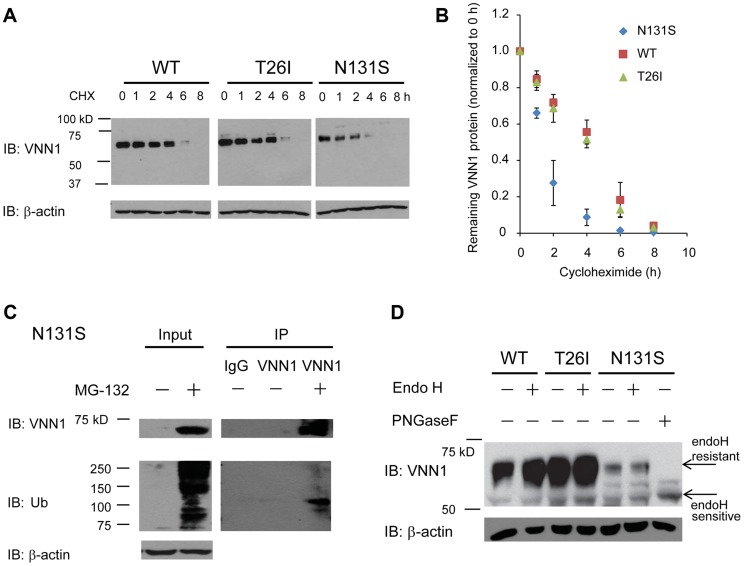
N131S Vanin-1 is subjected to rapid ERAD. HEK293 cells stably expressing WT, T26I or N131S vanin-1 were generated using the G418 selection method. (**A**) Cells were treated with cycloheximide (CHX, 100 µg/ml) for the indicated hours before being lysed. 30 µg of total protein were subjected to 8% SDS-PAGE and Western blot analysis using a rabbit polyclonal anti-vanin-1 antibody (Pierce antibodies) (*n* = 3). β-actin serves as a loading control. The protein band intensity was quantified using ImageJ software from the NIH, shown in (**B**). Data is reported as mean ± SEM. (**C**) Cells expressing N131S vanin-1 were treated with MG-132 (5 µM, 24 h), a potent proteasome inhibitor, before being lysed. Cell lysates were immunoprecipitated using an anti-vanin-1 antibody and subjected to Western blot analysis. IgG was used a negative control for nonspecific binding during immunoprecipitation (lane 3). IP: immunoprecipitation. Ub: ubiquitin. (**D**) Cell lysates were digested with endoglycosidase H (endoH) enzyme before Western blot analysis. Peptide-N-glycosidase F (PNGase F) cleaves the protein at a location between the innermost GlcNAc and asparagine residues from *N*-linked glycoproteins, serving as a control for unglycosylated vanin-1. EndoH resistant vanin-1 proteins fold properly in the ER and traffic at least through the Golgi. EndoH sensitive vanin-1 proteins are immature ER vanin-1 glycoform.

To determine the mechanism of loss of surface N131S vanin-1, we sought to confirm that N131S vanin-1 is rapidly degraded. A cycloheximide (CHX) chase assay was used to quantify the half-life of vanin-1 variants in HEK293 cells: WT vanin-1 had a half-life of 240 min; T26I vanin-1, 232 min; N131S vanin-1, 76 min, respectively (**Figure 4A**, quantification in **Figure 4B**). Thus, N131S vanin-1 has a much faster degradation rate than WT vanin-1, whereas T26I vanin-1 is degraded at a rate similar to that of WT vanin-1.

To confirm that misfolded N131S vanin-1 is subjected to ERAD, we applied MG-132 to the cells, which is a potent proteasome inhibitor. MG-132 treatment resulted in the accumulation of ubiquitinated proteins and substantially more total vanin-1 proteins (**Figure 4C**, cf. lane 2 to lane 1), indicating that efficient proteasome inhibition prevents the degradation of N131S vanin-1. Furthermore, using immunoprecipitation against vanin-1, we confirmed that MG-132 treatment resulted in ubiquitination of N131S vanin-1 (**Figure 4C**, cf. lane 5 to lane 4). These data indicate that N131S vanin-1 is subjected to rapid ERAD, resulting in loss of functional vanin-1 on the plasma membrane.

We hypothesize that rapid degradation of N131S vanin-1 resulted from its misfolding in the endoplasmic reticulum (ER). The endoglycosidase H (endo H) enzyme selectively cleaves vanin-1 after asparaginyl-*N*-acetyl-D-glucosamine (GlcNAc) in the *N*-linked glycans incorporated in the ER. After the high-mannose form is enzymatically remodeled in the Golgi, endo H is unable to remove the oligosaccharide chain. Therefore, endo H-resistant vanin-1 bands (with higher molecular weight) represent properly folded, post-ER vanin-1 glycoforms, which traffic at least to the Golgi compartment. The N131S mutation resulted in much less intense endo H-resistant bands than WT vanin-1 (**Figure 4D**, cf. lane 6 to lane 2), whereas T26I did not (**Figure 4D**, cf. lane 4 to lane 2). The ratio of endo H-resistant to total vanin-1 serves as a measure of vanin-1 trafficking efficiency. The trafficking efficiency of N131S vanin-1 was less than WT vanin-1, indicating that N131S vanin-1 does not fold properly in the ER. These data support the conclusion that N131S vanin-1 is misfolded in the ER and subsequently degraded by the ERAD pathway.

### HTN drugs decrease endogenous vanin-1 protein level

**Figure 5:**
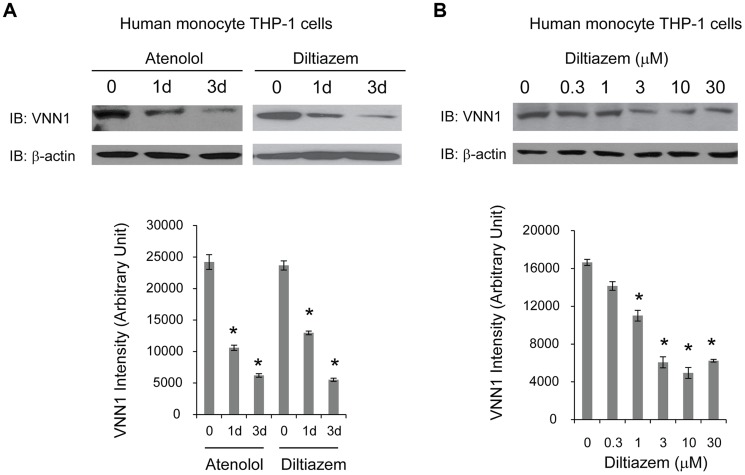
HTN drugs decrease endogenous vanin-1 protein level. Human blood-derived monocyte THP-1 cells were treated with diltiazem (10 µM), or atenolol (10 µM) for the indicated time (**A**) or treated with diltiazem for 3d using the indicated concentrations (**B**) before being lysed for SDS-PAGE and Western blot analysis using a rabbit polyclonal anti-vanin-1 antibody (*n* = 3). β-actin serves as a loading control. The protein band intensity was quantified using ImageJ software from the NIH, shown at the bottom. Data are reported as mean ± SEM. * *p*<0.05.

To determine whether vanin-1 is a target of current anti-hypertensive drugs, we tested the effect of two commonly prescribed HTN drugs with different known drug mechanisms on endogenous vanin-1 protein level. Human monocyte THP-1 cells were used because they were derived from human blood and have high endogenous WT vanin-1 protein expression levels. Two HTN drugs used are diltiazem [37], an L-type calcium channel blocker, and atenolol, a selective β1 adrenergic receptor blocker [38]. Treatment of THP-1 cells with diltiazem (10 µM) or atenolol (10 µM) for 1d or 3d decreased the endogenous total vanin-1 protein significantly in a time-dependent manner (**Figure 5A**, quantification shown below). Furthermore, application of diltiazem for 3d decreased the endogenous total vanin-1 protein significantly in a dose-dependent manner (**Figure 5B**, quantification shown below). This indicates that vanin-1 is a molecular target of current HTN drugs, which was previously unknown and confirms the relevance of vanin-1 to the regulation of blood pressure. Therefore, exploring other compounds that decrease vanin-1 level may lead to discovery of novel antihypertensive drugs, especially those with previously unknown function in HTN.

## Discussion

A major methodological issue that has greatly increased the challenges faced in the genetic epidemiology of BP is the high noise-to-signal ratio in the phenotype. This problem has numerous causes, including variation in measurement protocols of SBP and DBP across studies, the dynamic nature of BP levels, and concurrent use of antihypertensive medications. In addition, as with all polygenic disorders, the effect size for any single gene variant is very small and a large number of genes/variants are involved [10]. Recent large-scale BP genome-wide association studies (GWAS) of European, Asian and African ancestry populations demonstrated that the identified genetic variants together explain only 1–2% of BP variation [29], [39], [40]. It is thus not surprising that a large sample size is often necessary to detect genome-wide significant effects.

An analysis method complementary to GWAS is admixture mapping, which has been successfully applied to detect BP loci [11], [12], [41]. Our group reported that the missense variant rs2272996 (N131S) in *VNN1* was associated with BP through admixture mapping, and we conducted a follow-up association analysis in African and Mexican American samples [11], [12]. The association evidence in European-ancestry population is however less convincing [12], [13] In the current study, we performed meta-analysis using the COGENT consortium consisting of 19 studies with a total sample size of nearly 30,000 African ancestry subjects and confirmed the association evidence between rs2272996 and SBP (P = 0.01, **Table 1**).

However, statistical evidence alone cannot explain the role of a given variant on disease risk and drug response. Therefore, in our study we decided to analyze the functional effects of the N131S variant. Vanin-1 is a pantetheinase generating cysteamine, which regulates the glutathione-dependent oxidative stress response. We showed that the HTN-associated N131S mutation in vanin-1 significantly reduces vanin-1 total and cell surface expression. Consequently, the N131S vanin-1 only has fractional pantetheinase activity on the plasma membrane, which is associated with decreased HTN risk. Our result is consistent with the recognized link between impaired reduction-oxidation status and the development of HTN [19]–[21], and the observed protective effects in vanin-1^−/−^ mice in a variety of diseases, including oxidative stress [26], intestinal inflammation [27], and colon cancer [28], mostly due to higher glutathione storage to maintain a more reducing environment.

We further tested the drug effects of atenolol and diltiazem in human monocyte THP-1 cells, which have high endogenous WT vanin-1 protein expression level. Atenolol is a selective β1 adrenergic receptor blocker and developed as a replacement for propranolol in treating hypertension; diltiazem is a nondihydropyridine member of calcium channel blockers used in treatment of hypertension. We found that both drugs reduce the vanin-1 protein level in the THP-1 cells. The anti-hypertensive drugs may have different and complex mechanisms leading to reduced BP, but whether vanin-1 is targeted was previously unknown. Our experiments filled this gap and showed vanin-1 is involved in the BP regulation pathway. Therefore, other potent vanin-1 inhibitors may prove to have BP reducing effects, which is especially useful given that these inhibitors have not been studied in HTN and thus may provide new therapeutics for HTN.

Our study presented the first functional studies of vanin-1 in HTN association, and provides compelling evidence for the essential role of its N131S mutation. Nonetheless, it has been demonstrated that multiple variants in a gene may contribute to a phenotypic variation [42], [43], and it is possible that other closely linked variants may have similar or analogous effects, or act in combination with N131S to regulate the vanin-1 protein expression and function. Current GWAS of HTN related traits mainly focus on testing common variants (MAF: minor allele frequency ≥5%) through pre-built chips and imputations based on HapMap [44] data; to date those findings in general have modest effect sizes [39]. Other functional and rare variants may be identified by deep sequencing, in combination with publicly available databases, such as the 1000 Genome Projects [45] and the Encyclopedia of DNA Elements (ENCODE) [46]. Identification of additional functional SNPs in *VNN1* and their association with BP should provide further evidence for vanin-1 function in the regulation of BP.

Cell lines were used to determine that ERAD is the underlying mechanism for vanin-1's loss of function due to the N131S mutation. Cell lines are commonly used for the study of molecular mechanisms because they typically provide efficient transfection and a physiologically-relevant cell environment for the target protein. However, BP has a complex etiology with the involvement of a variety of organs, such as heart, brain and kidney, which cannot be recapitulated solely in cell lines. Although knowledge gained from our cell system provides essential cellular mechanistic insights into the regulation of vanin-1 and its function, the study of BP regulation by vanin-1 calls for studies in animal models. A hypertensive mouse or rat model, vanin-1 knockout mouse or rat model, and N131S vanin-1 knockin mouse or rat model would be of great interest to study the effects of vanin-1 and its mutation in the complex physiological and metabolic systems.

Vanin-1 provides a potential candidate to be manipulated to ameliorate HTN. Vanin-1 is a pantetheinase that contains the conserved catalytic triad residue–glutamate, lysine and cysteine–within the nitrilase family [47]. Based on the sequence alignment of vanin-1 with other nitrilase family members, the conserved catalytic triad of vanin-1 is composed of glutamate 79, lysine 178 and cysteine 211 [23]. A three-dimensional atomic model of vanin-1 was built using the I-TASSER server (**Figure S3**) [48]. Neither T26 nor N131 is in the vicinity of the catalytic sites of vanin-1. Therefore, the T26I and N131S mutations *per se* are not expected to change the vanin-1 enzyme activity significantly. Indeed, we showed that the T26I mutation did not influence vanin-1 maturation or enzymatic activity. The N131S mutation has much weaker pantetheinase activity, presumably due to exceedingly low concentration of N131S vanin-1 on the plasma membrane; however, the activity is still evident, implying that the catalytic triad is not disrupted by this mutation.

Loss of function of vanin-1 is caused by misfolding and rapid degradation of vanin-1 due to a single missense mutation from Asn to Ser at position 131. As a GPI-anchored protein, to function properly, vanin-1 needs to be trafficked efficiently to the plasma membrane, where it acts as a pantetheinase. In accordance with the maturation of general GPI-anchored proteins [15], vanin-1 is co-translationally translocated into the ER for folding. Because human vanin-1 has six potential *N*-linked glycosylation sites, its maturation is presumably dictated by glycoprotein processing machinery in the ER [49], [50]. Properly folded vanin-1 is trafficked out of the ER, through the Golgi and to the plasma membrane in a fully functional state. Misfolded vanin-1 is recognized by the ER quality control machinery and subjected to ERAD, being retrotranslocated to the cytosol, ubiquitinated and degraded by the proteasome [51]–[54]. Cells need to maintain a delicate balance between protein synthesis, folding, trafficking, aggregation and degradation for individual proteins that make up the proteome in normal physiology. This balance is dictated by the cellular protein homeostasis (proteostasis) network, composed of a variety of sub-networks, including the chaperone, degradation and trafficking networks, and cellular signaling pathways that regulate proteostasis as the core layers [55]–[57]. Therefore, further elucidation of the proteostasis network for vanin-1 should provide a valuable fine-tuning control of vanin-1 expression, function and BP.

## Materials and Methods

### Sample cohorts

19 cohort studies contributed to the meta-analysis of BP and genetic variants in *VNN1* in African-Americans as detailed in Franceschini et al [29], including Biological Bank of Vanderbilt University (BioVU); Atherosclerosis Risk In Communities (ARIC); Coronary Artery Risk Development in Young Adults (CARDIA); Cleveland Family Study (CFS); Jackson Heart Study (JHS); Multi-Ethnic Study of Atherosclerosis (MESA); Cardiovascular Health Study (CHS); Genetic Study of Atherosclerosis Risk (GeneSTAR); Genetic Epidemiology Network of Arteriopathy (GENOA); The Healthy Aging in Neighborhoods of Diversity Across the Life Span Study (HANDLS); Health, Aging, and Body Composition (Health ABC) Study; The Hypertension Genetic Epidemiology Network (HyperGEN); Mount Sinai, New York City, USA Study (Mt Sinai Study); Women's Health Initiative SNP Health Association Resource (WHI); Howard University Family Study (HUFS); Bogalusa Heart Study (Bogalusa); Sea Islands Genetic Network (SIGNET); Loyola Maywood Study (Maywood); and Loyola Nigeria Study (Nigeria). Each study received IRB approval of its consent procedures, examination and surveillance components, data security measures, and DNA collection and its use for genetic research.

### The vanin-1 expression in human plasma samples

We selected 24 plasma samples from the International Collaborative Study on Hypertension in Blacks (ICSHIB), in which the study participants were recruited from Igbo-Ora and Ibadan in southwest Nigeria as part of a long-term study on the environmental and genetic factors underlying hypertension [58]. The ICSHIB included 1,188 subjects who were genotyped using Affymetrix platform 6.0 chip [59]. We selected 6 subjects per group from the high and lower SBP traits in each of TT and CC genotype groups of SNP rs2272996. For each of these 24 subjects, western blot analysis was performed by controlling the same amount of total plasma protein.

### Statistical analysis

The detailed statistical analysis of each cohort can be found in Franceschini et al [29]. In brief, each study cohort received a uniform statistical analysis protocol and analyses were conducted accordingly. BP was measured in mmHg. For individuals reporting use of antihypertensive medications, BP was imputed by adding 10 and 5 mmHg for SBP and DBP, respectively. For unrelated individuals, SNP associations for SBP or DBP were assessed by linear regression assuming an additive model, adjusting for age, age^2^, body mass index (BMI) and gender. Population stratification was controlled by adjusting for the first 10 principal components obtained from selected ancestry informative markers [60], [61]. For family data, association was tested using a linear mixed effect model, where random effects account for family structure [62].

Meta-analysis across the 19 cohorts was performed by applying both fixed-effect [31], [32] and random-effect [33] models to estimate the overall effect. The fixed-effect model assumes that the effect size is the same for all the included studies; the only source of error is the random error within studies, which depends primarily on the sample size for each study. Because the inverse variance is roughly proportional to sample size, the fixed-effect model provides a weighted average of the effect sizes, with the weights being the estimated inverse of the variance of the estimate in each study. The random-effect model assumes that the effect sizes from studies are similar but not identical, dependent on each study protocol; the source of error includes within-study and among-study error [33]. It is more conservative and thus provides relatively wider 95% confidence intervals when heterogeneity across studies exists.

All experimental data are presented as mean ± SEM, and any statistical significance was calculated using two-tailed Student's *t*-test.

### Reagents

MG-132, diltiazem, and atenolol were obtained from Sigma-Aldrich. The pCMV6 plasmids containing human vanin-1 and pCMV6 Entry Vector plasmid (pCMV6-EV) were obtained from Origene. The human vanin-1 missense mutations, N131S and T26I, were constructed using QuickChange II site-directed mutagenesis Kit (Agilent Genomics), and the cDNA sequences were confirmed by DNA sequencing, showing the single-site mutation of these variants. The rabbit polyclonal anti-vanin-1 antibody came from Pierce antibodies, the mouse monoclonal anti-transferrin antibody from Santa Cruz Biotechnology, the mouse monoclonal anti-β-actin antibody from Sigma, and the rabbit polyclonal anti-ubiquitin antibody from Cell Signaling.

### Cell culture and transfection

Human embryonic kidney 293 (HEK293) cells and human monocytic THP-1 cells came from ATCC. THP-1 cells were maintained in RPMI-1640 medium (Hyclone) with 10% heat-inactivated fetal bovine serum (Sigma-Aldrich) and 1% Pen-Strep (Hyclone) at 37°C in 5% CO_2_. HEK293 cells were maintained in Dulbecco's Modified Eagle Medium (DMEM) (Hyclone) with 10% heat-inactivated fetal bovine serum (Sigma-Aldrich) and 1% Pen-Strep (Hyclone) at 37°C in 5% CO_2_. Monolayers were passaged upon reaching confluency with TrypLE Express (Life Technologies). HEK293 cells were grown in 6-well plates or 10-cm dishes and allowed to reach ∼70% confluency before transient transfection using Lipofectamine 2000 (Life Technologies) according to the manufacturer's instruction. Stable cell lines expressing vanin-1 variants (WT, N131S or T26I) were generated using the G-418 selection method. Briefly, transfected cells were maintained in DMEM supplemented with 0.8 mg/mL G418 (Enzo Life Sciences) for 15 days. G-418 resistant cells were selected for follow-up experiments.

### Western blot analysis

Cells were harvested and then lysed with lysis buffer (50 mM Tris, pH 7.5, 150 mM NaCl, and 1% Triton X-100) supplemented with Roche complete protease inhibitor cocktail. Lysates were cleared by centrifugation (15,000× *g*, 10 min, 4°C). Protein concentration was determined by MicroBCA assay (Pierce). Endoglycosidase H (endo H) or Peptide-N-Glycosidase F (PNGase F) (New England Biolabs) enzyme digestion was performed according to published procedure [35]. Aliquots of cell lysates or human plasma samples were separated in an 8% SDS-PAGE gel, and Western blot analysis was performed using the appropriate antibodies. Band intensity was quantified using Image J software from the NIH.

### Biotinylation of cell surface proteins

HEK293 cells stably expressing vanin-1 variants were plated in 10-cm dishes for surface biotinylation experiments according to published procedure [35]. Intact cells were washed twice with ice-cold PBS and incubated with the membrane-impermeable biotinylation reagent Sulfo-NHS SS-Biotin (0.5 mg/mL; Pierce) in PBS containing 0.1 mM CaCl_2_ and 1 mM MgCl_2_ (PBS+CM) for 30 min at 4°C to label surface membrane proteins. To quench the reaction, cells were incubated with 10 mM glycine in ice-cold PBS+CM twice for 5 min at 4°C. Sulfhydryl groups were blocked by incubating the cells with 5 nM N-ethylmaleimide (NEM) in PBS for 15 min at room temperature. Cells were solubilized for 1 h at 4°C in lysis buffer (Triton X-100, 1%; Tris–HCl, 50 mM; NaCl, 150 mM; and EDTA, 5 mM; pH 7.5) supplemented with Roche complete protease inhibitor cocktail and 5 mM NEM. The lysates were cleared by centrifugation (16,000× g, 10 min at 4°C) to pellet cellular debris. The supernatant contained the biotinylated surface proteins. The concentration of the supernatant was measured using microBCA assay (Pierce). Biotinylated surface proteins were affinity-purified from the above supernatant by incubating for 1 h at 4°C with 100 µL of immobilized neutravidin-conjugated agarose bead slurry (Pierce). The samples were then subjected to centrifugation (16,000×g, 10 min, at 4°C). The beads were washed six times with buffer (Triton X-100, 0.5%; Tris–HCl, 50 mM; NaCl, 150 mM; and EDTA, 5 mM; pH 7.5). Surface proteins were eluted from beads by boiling for 5 min with 60 µL of LSB/Urea buffer (2× Laemmli sample buffer (LSB) with 100 mM DTT and 6 M urea; pH 6.8) for SDS-PAGE and Western blotting analysis.

### Cell-based fluorescence assay to evaluate vanin-1 variants' pantetheinase activity

The cell-based fluorescence assay to evaluate vanin-1's pantetheinase activity was performed according to published procedure with modifications [36]. The substrate, pantothenate-7-amino-4-methylcoumarin (pantothenate-AMC) was chemically synthesized according to published method [36]. As a pantetheinase, vanin-1 catalyzed the release of AMC, giving a fluorescence signal at excitation 350 nm and emission 460 nm. HEK293 cells expressing vanin-1 variants were lysed with lysis buffer (50 mM Tris, pH 7.5, 150 mM NaCl, and 1% Triton X-100) supplemented with Roche complete protease inhibitor cocktail. Enzyme activity was performed using 10 µg of total proteins containing the substrate pantothenate-AMC (5 µM), 0.5 mM DTT, 5% DMSO in a 100 µL final volume in PBS, pH 7.5. Fluorescence signals at excitation 350 nm and emission 460 nm measuring the released AMC were recorded every 3 min at 37°C in 96-well plates (Greiner Bio-One) using a fluorescence plate reader. A 60-min kinetic assay in four replicates and three biological replicates was carried out. Buffer only and HEK293 cells transfected with empty vector (EV) were used as negative controls for non-specific pantetheinase activity.

### Cycloheximide (CHX) chase assay

HEK293 cells stably expressing vanin-1 variants were seeded at 2.5×10^5^ cells per well in 6-well plates and incubated at 37°C overnight. To stop protein translation, cells were treated with 100 µg/mL cycloheximide (Ameresco) and chased for the indicated time. Cells were then lysed for SDS-PAGE and Western blot analysis.

### Immunoprecipitation

Cell lysates (500 µg) were pre-cleared with 30 µL of protein A/G plus-agarose beads (Santa Cruz) and 1.0 µg of normal rabbit IgG for 1 hour at 4°C to remove nonspecific binding proteins [63]. The pre-cleared cell lysates were incubated with 2.0 µg of rabbit anti-vanin-1 antibody (Pierce) for 1 hour at 4°C, and then with 30 µL of protein A/G plus agarose beads overnight at 4°C. The beads were collected by centrifugation at 8000×g for 30 s, and washed four times with lysis buffer. The vanin-1 protein complex was eluted by incubation with 30 µL of SDS loading buffer in the presence of 100 mM DTT. The immunopurified eluents were separated in 8% SDS-PAGE gel, and Western blot analysis was performed.

## Supporting Information

Figure S1 Descriptive characteristics of SBP (A), DBP (B), age (C) and BMI (D) for the 19 COGENT consortium cohorts. The mean and SD are presented for each cohort.
(PDF)

Figure S2 Antihypertensive medication rates for the 19 COGENT consortium cohorts.
(PDF)

Figure S3 Homology model for vanin-1 protein. The vanin-1 three-dimensional atomic model was built using I-TASSER server (http://zhanglab.ccmb.med.umich.edu/I-TASSER) [48]. The predicted β-sheet regions are in red; α-helix, cyan; loop, purple. The putative catalytic triad residues (E78, K178 and C211) are shown as a sphere model. T26 and N131 are shown as a stick model.
(PDF)

Table S1 The allele frequency of rs2272996 in the different studies of the COGENT consortium.
(DOCX)

Table S2 Meta-analysis results of the replication cohort data for SNP rs7739368.
(DOCX)
