# Supplementary figures and images for: The Association of the Vanin-1 N131S Variant with Blood Pressure Is Mediated by Endoplasmic Reticulum-Associated Degradation and Loss of Function
Source: PLoS Genet. 2014 Sep 18;10(9):e1004641. doi: 10.1371/journal.pgen.1004641 (PMC4169380; doi:10.1371/journal.pgen.1004641)

A

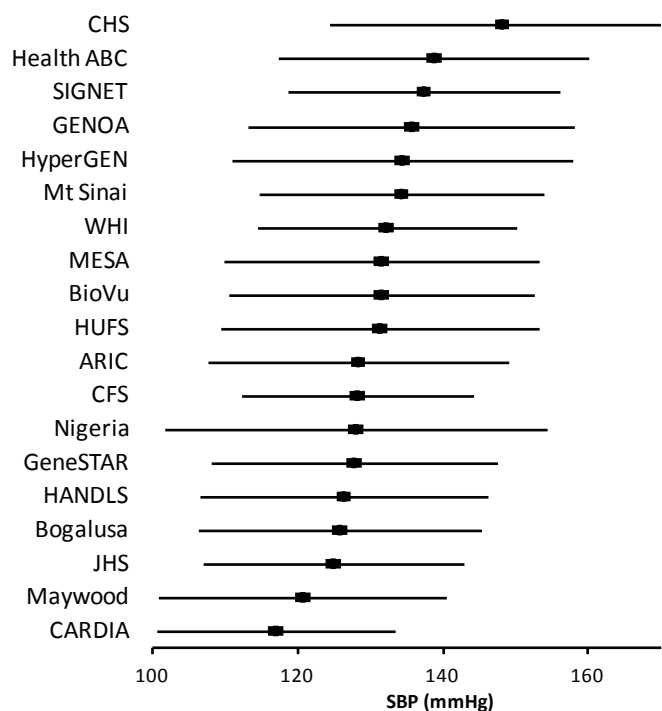

B

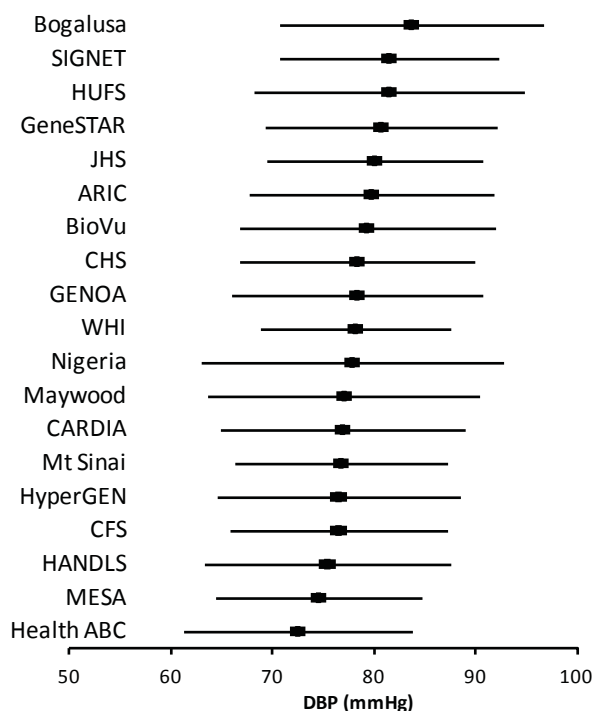

C

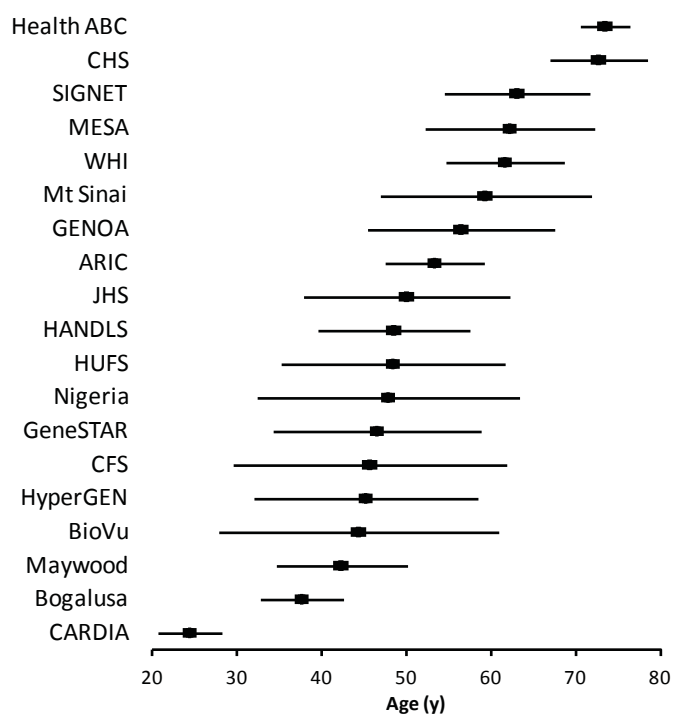

D

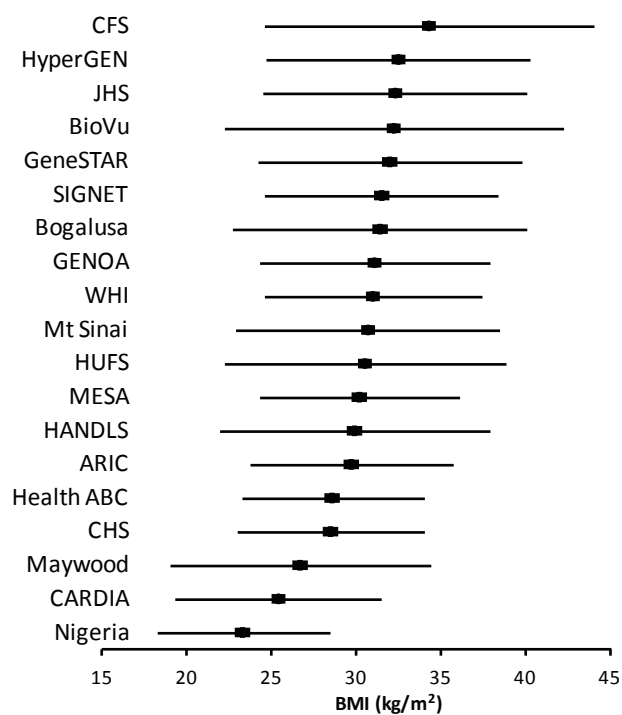

Figure S1

Supplement: Figure S1 — Descriptive characteristics of SBP (A), DBP (B), age (C) and BMI (D) for the 19 COGENT consortium cohorts. The mean and SD are presented for each cohort. (PDF) [file pgen.1004641.s001.pdf]

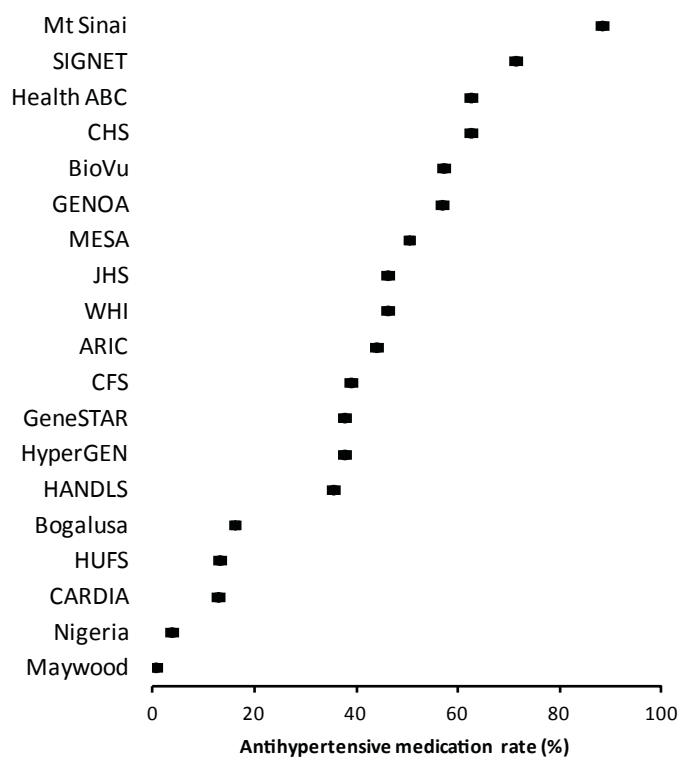

**Figure S2**

Supplement: Figure S2 — Antihypertensive medication rates for the 19 COGENT consortium cohorts. (PDF) [file pgen.1004641.s002.pdf]

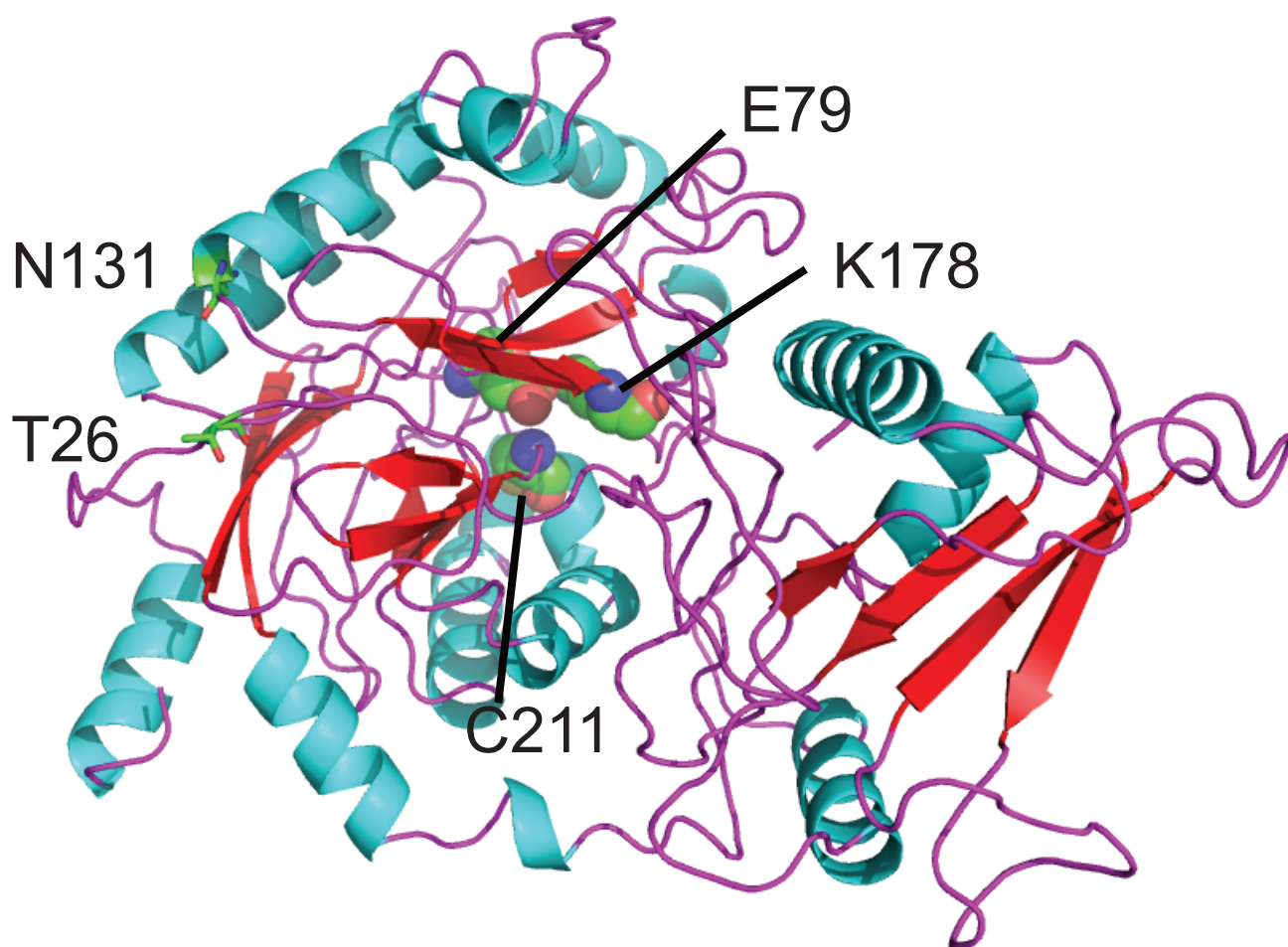

**Figure S3**

Supplement: Figure S3 — Homology model for vanin-1 protein. The vanin-1 three-dimensional atomic model was built using I-TASSER server (http://zhanglab.ccmb.med.umich.edu/I-TASSER) [48]. The predicted β-sheet regions are in red; α-helix, cyan; loop, purple. The putative catalytic triad residues (E78, K178 and C211) are shown as a sphere model. T26 and N131 are shown as a stick model. (PDF) [file pgen.1004641.s003.pdf]
