# Supplementary material for: The Association of the Vanin-1 N131S Variant with Blood Pressure Is Mediated by Endoplasmic Reticulum-Associated Degradation and Loss of Function
Source: PLoS Genet. 2014 Sep 18;10(9):e1004641. doi: 10.1371/journal.pgen.1004641 (PMC4169380; doi:10.1371/journal.pgen.1004641)
Supplement: Table S1 — The allele frequency of rs2272996 in the different studies of the COGENT consortium. (DOCX) [file pgen.1004641.s004.docx]

**Table S1**. The allele frequency of rs2272996 in the different studies of the COGENT consortium. AF, allele frequency in terms of reference allele T.

| Cohort | AF |
| --- | --- |
| CHS | 0.7862 |
| HANDLS | 0.7899 |
| SIGNET | 0.8010 |
| CFS | 0.8019 |
| HUFS | 0.8034 |
| WHI | 0.8034 |
| ARIC | 0.8078 |
| MtSinai | 0.8090 |
| MESA | 0.8099 |
| GENOA | 0.8104 |
| JHS | 0.8107 |
| Health ABC | 0.8108 |
| CARDIA | 0.8170 |
| HyperGEN | 0.8172 |
| Maywood | 0.8200 |
| BioVU | 0.8292 |
| Nigeria | 0.8350 |
| Bogalusa | 0.8496 |
