# Supplementary material for: The Association of the Vanin-1 N131S Variant with Blood Pressure Is Mediated by Endoplasmic Reticulum-Associated Degradation and Loss of Function
Source: PLoS Genet. 2014 Sep 18;10(9):e1004641. doi: 10.1371/journal.pgen.1004641 (PMC4169380; doi:10.1371/journal.pgen.1004641)
Supplement: Table S2 — Meta-analysis results of the replication cohort data for SNP rs7739368. (DOCX) [file pgen.1004641.s005.docx]

**Table S2**. Meta-analysis results of the replication cohort data for SNP rs7739368. The effect size (beta) is presented in terms of the reference allele A1 for the SNP of interest. SE, standard error.

|  |  |  |  | Direction in each cohort | Heterogeneity *p* value | Fixed-effects Model | | |  | Random-effects Model | | |
| --- | --- | --- | --- | --- | --- | --- | --- | --- | --- | --- | --- | --- |
| SNP | Phenotype | A1 | Frequency |  |  | Beta (95% CI) | SE | *p* value |  | Beta (95% CI) | SE | *p* value |
| rs7739368 | SBP | A | 0.88 | +?-++-+-+++-+--+-+++ | 0.73 | 0.68 (0.22, 1.14) | 0.23 | 0.004 |  | 0.68 (0.22, 1.14) | 0.23 | 0.004 |
